# Investigation of the solubility and thermodynamics of salicylic acid in 2-propanol and ethylene glycol/propylene glycol binary solvent mixtures at (293.15 to 313.15) K

**DOI:** 10.1186/s13065-024-01188-1

**Published:** 2024-05-09

**Authors:** Fatemeh Dabagh, Fatemeh Khalili, Aynaz Zarghampour, Salar Hemmati, Jalal Hanaee, Elaheh Rahimpour, Abolghasem Jouyban

**Affiliations:** 1 Student Research Committee, Tabriz University of Medical Sciences, Tabriz, Iran; 2 Pharmaceutical Analysis Research Center, Faculty of Pharmacy, Tabriz University of Medical Sciences, Tabriz, Iran; 3 Drug Applied Research Center, Tabriz University of Medical Sciences, Tabriz, Iran; 4 Research Center for Pharmaceutical Nanotechnology, Tabriz University of Medical Sciences, Tabriz, Iran; 5 Infectious and Tropical Diseases Research Center, Tabriz University of Medical Sciences, Tabriz, Iran

**Keywords:** Solubility, Salicylic acid, Thermodynamic parameters, Glycols

## Abstract

This study aimed to measure both the solubility and thermodynamics of salicylic acid in binary solvent mixtures of (2-propanol + ethylene glycol) and (2-propanol + propylene glycol) at different temperatures in the range of 293.2–313.2 K. The experimental solubility data were analyzed using various linear and nonlinear cosolvency models, such as the van’tt Hoff, Jouyban-Acree, Jouyban-Acree-van’tt Hoff, mixture response surface and modified Wilson models and to evaluate the models, the mean relative deviations of the back-calculated solubility data were compared with experimental values. Through this analysis, the apparent thermodynamic parameters, including Gibbs energy, enthalpy, and entropy were calculated using the van’tt Hoff and Gibbs equations for this system. Additionally, the density values for salicylic acid saturated mixtures were also measured and represent mathematically using the Jouyban-Acree model.

## Introduction

Salicylic acid (SA) or ortho-hydroxybenzoic acid is a type of phenolic and beta hydroxy acid that exists in various plants. It is a cyclooxygenase I and II inhibitor that reduces the level of prostaglandins and thromboxanes in the body. Hence, SA has also antioxidative activities [1] and its salts and esters (salicylates) have anti-inflammatory effects [2]. SA is important in the pharmaceutical industry as it is the precursor of the widely used drug aspirin. Aspirin, a trade title for acetylsalicylic acid, hydrolyzes naturally to SA. It also helps to treat skin conditions due to its exfoliating and comedolytic effects [3]. SA is undoubtedly a critical plant hormone that directs plant insusceptibility. Additionally, SA can direct distinctive reactions, such as B-Resilience to abiotic push, plant development and advancement, and soil microbiome [4]. SA is a compound that is sparingly soluble in water and well soluble in polar organic solvents [3].

Equilibrium solubility is included in several pharmaceutical process such as drug purification procedures, drug identification, and the design of homogeneous pharmaceutical dosage forms [5]. The information on solubility and dissolution is critical to the pharmaceutical field, since it licenses the researcher to choose the finest dissolution medium for a drug or drug combination, and helps to overcome particular troubles within the manufacture of pharmaceutical solutions [6]. There are several strategies to improve the solubility of drugs such as micronization, chemical modification, pH adjustment, solid dispersion, complexation, cosolvency, micellar solubilization, hydrotropic, etc [6]. . Cosolvency is one of the most common techniques that are easy to use and evaluate, and quick to formulate [7]. Until now, the solubility of SA was reported in the binary solvent mixtures of (methanol/ethanol/2-propanol/1-propanol) + water [8, 9], (polyethylene glycol 300/1, 4-dioxane) + water [9], propylene glycol (PG)/N-methylpyrrolidone (NMP) + ethanol [10], NMP + PG [10], polyethylene glycols 200, 400 and 600 + water [11], and betaine-based deep eutectic solvents + water [12]. However, no data have been reported for its solubility in 2-propanol + PG or ethylene glycol (EG) mixtures. It is crucial to emphasize that the presented study serves as an important contribution to a more extensive, interdisciplinary investigation focusing on the notable enhancement of SA’s solubility library. By examining the solubility in almost all commonly used solvents, the study expands our scientific understanding in this area, which may lead to substantial advancements in pharmaceutical and biomedical applications involving SA.

In this study, we determined the solubility of SA in two binary systems of (2-propanol + PG) and (2-propanol + EG) at varying temperatures in the range of 293.2–313.2 K using the cosolvency approach. We then correlated these results with different linear and non-linear cosolvency models. Additionally, we calculated the apparent dissolution thermodynamic properties for dissolution of SA in the investigated systems.

## Chemicals and methods

### Chemicals

SA (with a mass fraction purity of > 0.999 from Julian Kimia Sanat, Iran), PG (0.990%, from Merck, Germany), EG (0.995%, from Merck, Germany), 2-propanol (0.998%, from Merck, Germany) were used for the preparation of saturated solutions. Specifically, ethanol with a mass fraction purity of 0.935 (Jahan Alcohol Teb, Iran) and distilled water (lab-made) were employed for diluting the saturated SA solutions prior to UV-Vis measurements.

### Solubility determination of SA

**Fig. 1:**
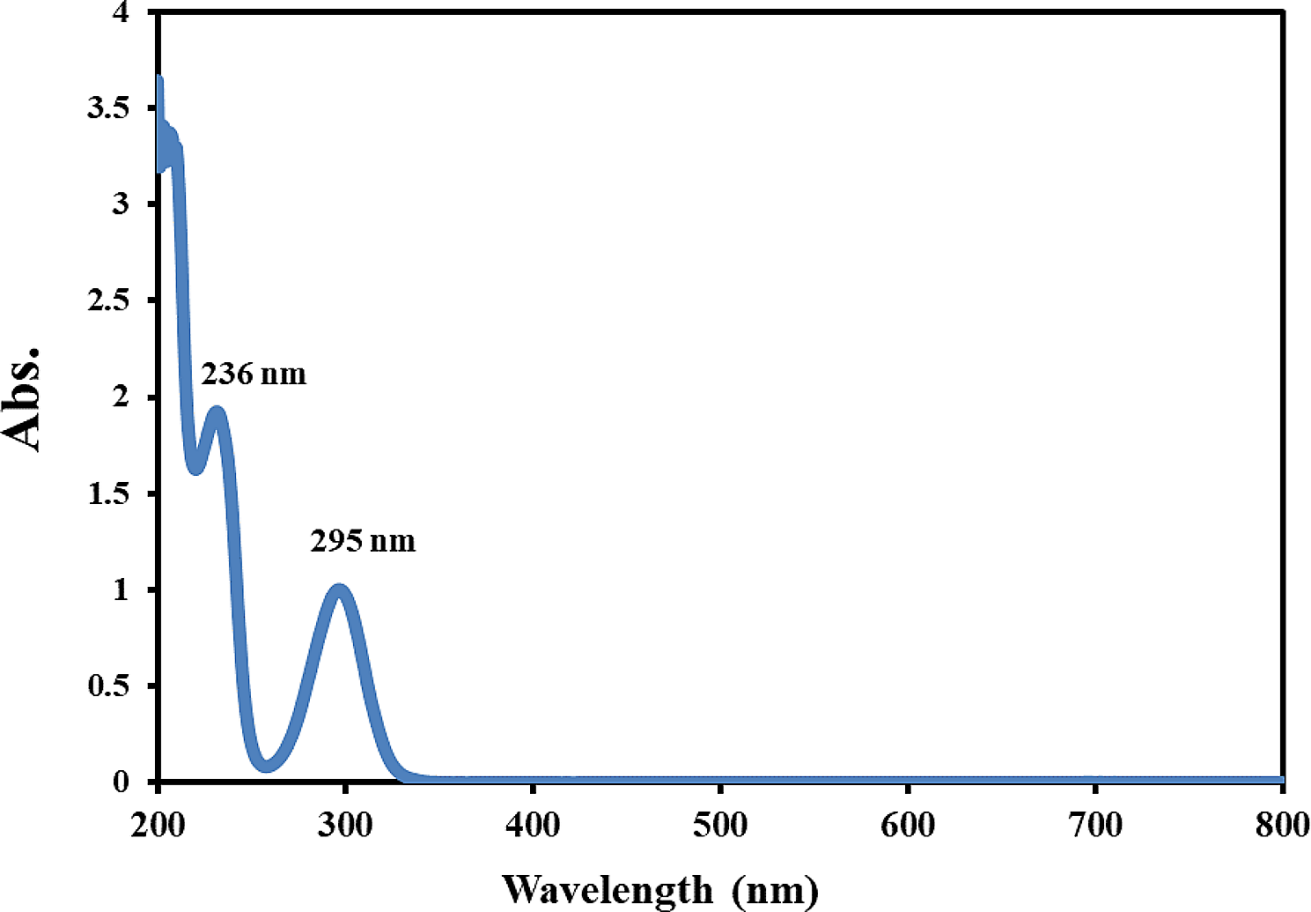
Absorbance spectrum of SA in the wavelength range of 200–800 nm

A shake-flask approach followed by UV-Vis spectroscopy method was used for the determination of SA solubility in binary mixtures of (2-propanol + PG) and (2-propanol + EG) [8]. To this end, an excess amount of SA was added to 7 mL tubes containing neat solvents and pre-mixed solvents of (2-propanol + PG) or (2-propanol + EG) with a total mass of 4.0 g. The mass fractions ranged from 0.1 to 0.9. Subsequently, sealed tubes were transferred to an incubator (by Nabziran Industrial Group, Tabriz, Iran) and subjected to continuous shaking (Behdad, Tehran, Iran) inside the incubator. The duration of incubation time was determined based on preliminary findings on dissolution rates. The system was allowed to reach a state of solid-liquid equilibrium over a period of 72 h, then the saturated mixtures were centrifuged, an aliquot of the clear upper solutions was taken and diluted in proper ratios. In the case of concentrated solutions, dilution was carried out using ethanol: water 50:50% (v/v). The concentration of SA was determined using a UV-Vis spectrophotometer (UV-1800 model, Shimadzu, Kyoto, Japan) by measuring the absorbance at 236 nm (Fig. 1). It should be noted that SA shows two distinct peak in the range of 200–800 nm (236 and 295 nm) which wavelength of 236 nm was chosen in this study due to high sensitivity. The density of the saturated solutions was determined using a 2 mL pycnometer and an analytical balance with a precision of 0.0001 g.

### X-ray powder diffraction (XRD) analysis

The crystalline structure of SA (in both raw and residual forms, dissolved in the investigated solvents) was examined using XRD analysis conducted on a PHILIPS PW1730 instrument. The XRD results were obtained within the range of 10° to 40° (2θ) at a current of 30 mA and voltage of 40 kV, under atmospheric pressure.

### Computation section

Some linear models (i.e., the van’tt Hoff, the Jouyban-Acree, the Jouyban-Acree-van’tt Hoff, the mixture response surface (MRS)) and a non-linear mathematical model (i.e., the modified Wilson) have been utilized to fit the experimental solubility values of SA in binary mixtures of 2-propanol and EG/PG. The main reason for selection of these models for correlation was based on our previous finding. However, in detail the van’tt Hoff model is a commonly used model for relating the solubility to temperature in the narrow of temperature ranges. Herein, solubility of SA was investigated in five temperatures with interval value of five degrees’ kelvin which can be accurately fitted to this model. The next model is MRS which relates the solubility to solvent composition. The model is chosen as a model linear equation for solubility-solvent composition function. Moreover, the modified Wilson is also chosen as a model non-linear equation for solubility-solvent composition function. The last models are the Jouyban-Acree and the Jouyban-Acree-van’tt Hoff which relate the solubility to both temperature and solvent composition. These models are chosen based on this important property which correlate/predict all gathered solubility data in various temperatures and solvent compositions in same equation with high quality for data predicting based on our previous reports. The specifications of every model are delineated in the following section. It should be noted that all computations were performed using simple linear or non-linear regressions in SPSS 16.0 software. About linear regression, the relationship between the dependent variable (*Y*) and the independent variables (*X*_*1*_, *X*_*2*_, *X*_*3*_, etc.) is represented by a linear equation:

*Y* = *β*_0_ + *β*_1_ × *X*_1_ + *β*_2_ × *X*_2_ + *β*_3_ × *X*_3_ + … + *ε*

The primary objective of linear regression is to estimate the coefficients (*β*_*0*_, *β*_*1*_, *β*_*2*_, *β*_*3*_, etc.) that best fit the data, minimizing the sum of squared errors between the predicted values (Y_pre_) and the actual values of Y. This is known as the least squares method. In non-linear regression, the relationship between *Y* and the independent variables is defined by a non-linear equation. Again, the goal is to estimate the parameters (coefficients) of this non-linear equation that best fit the data, minimizing the sum of squared errors between the predicted values (Y-hat) and the actual values of Y. Levenberg-Marquardt algorithm analyzes unconstrained models. This method consisted of three steps which all of them is performed in SPSS software. These steps are (i) Initialize with a get value by user, calculate residuals, and compute the Jacobian matrix. (ii) Update parameter values using the Gauss-Newton method (if applicable) or the steepest descent method. (iii) Check for convergence and adjust the scaling factor; repeat steps i-ii until convergence criteria are met [13].

### Van’t Hoff equation

The correlation between temperature and solubility data can be accurately described by the van’t Hoff equation [14]:

1 $$ \text{l}\text{n}x=A+\frac{B}{T}$$

The coefficients of this model are *A* and *B*.

### Jouyban-Acree model

The Jouyban-Acree model, which is a multiple linear cosolvency model, is commonly employed to correlate solubility data by establishing a relationship between solubility values, temperature, and the composition of the solvent. The equation takes a general form, and it is used to analyze solubility data for a wide range of compounds [15]. Furthermore, the Jouyban-Acree model can also be used to correlate SA density measurements in the mixture of 2-propanol + EG and 2-propanol + PG.

2 $$ {\text{l}\text{n}x}_{m,T}={w}_{1}{\text{l}\text{n}x}_{1,T}+{w}_{2}{\text{l}\text{n}x}_{2,T}+\frac{{w}_{1}{w}_{2}}{T}\sum _{i=0}^{2}{J}_{i}{({w}_{1}-{w}_{2})}^{i}$$

where *x*_1,*T*_ and *x*_2,*T*_ are mole fraction drug solubilities in mono-solvents 1 and 2, *x*_*m, T*_ is the drug solubility in the solvent mixture at temperature *T*. The *J*_*i*_ parameters are obtained by linear regression analysis of ${\text{l}\text{n}x}_{m,T}-{w}_{1}{\text{l}\text{n}x}_{1,T}-{w}_{2}{\text{l}\text{n}x}_{2,T}$ against $\frac{{w}_{1}{w}_{2}}{T}$, $\frac{{w}_{1}{w}_{2}({w}_{1}-{w}_{2})}{T}$, $\frac{{w}_{1}{w}_{2}{({w}_{1}-{w}_{2})}^{2}}{T}$.

### Jouyban-Acree-Van’t Hoff model

An accurate method for predicting/correlating drug solubility in solution mixtures can be achieved by combining Jouyban-Acree with van’t Hoff according to Jouyban-Acree-van’t Hoff’s equation (Eq. (3)) [15].

3 $$ {\text{l}\text{n}x}_{m,T}={w}_{1}\left({A}_{1}+\frac{{B}_{1}}{T}\right)+{w}_{2}\left({A}_{2}+\frac{{B}_{2}}{T}\right)+\frac{{w}_{1}{w}_{2}}{T}\sum _{i=0}^{2}{J}_{i}{({w}_{1}-{w}_{2})}^{i}$$

*A*_1_, *B*_1_, *A*_2_ and *B*_2_ are the van’t Hoff model’s constants (intercept and slope) obtained by plotting $\text{l}\text{n}{x}_{m,T}$ against 1/*T* in the mono-solvents at various temperatures. *J*_*i*_ terms are computed using linear regression of $(\text{l}\text{n}{x}_{m,T}-{w}_{1}\left({A}_{1}+\frac{{B}_{1}}{T}\right)-{w}_{2}\left({A}_{2}+\frac{{B}_{2}}{T}\right))$ vs. $\frac{{w}_{1}.{w}_{2}}{T}$, $\frac{{w}_{1}.{w}_{2}({w}_{1}-{w}_{2})}{T}$, and$\frac{ {w}_{1}.{w}_{2}{({w}_{1}-{w}_{2})}^{2}}{T}$.

### MRS model

As another linear model, MRS correlates the solubility data at isothermal conditions as follows [16]:

4 $$ {\text{l}\text{n} x}_{m}={\beta }_{1}{w}_{1}^{{\prime }}+{\beta }_{2}{w}_{2}^{{\prime }}+{\beta }_{3}\left(\frac{1}{{w}_{1}^{{\prime }}}\right)+{\beta }_{4}\left(\frac{1}{{w}_{2}^{{\prime }}}\right)+{\beta }_{5}{w}_{1}^{{\prime }}{w}_{2}^{{\prime }}$$

${\beta }_{1}-{\beta }_{2}$ are the parameters of the present equation, and the ${w}_{1}^{{\prime }}$ and ${w}_{2}^{{\prime }}$ are obtained as follows: ${w}_{1}^{{\prime }}=0.96{w}_{1}+0.02$ and ${w}_{2}^{{\prime }}=0.96{w}_{2}+0.02$.

### The modified Wilson model

To obtain the drug solubility in binary mixed solvents at isothermal conditions, a non-linear model of the modified Wilson is also used [17]. Its general form is as:

5 $$ -{\text{l}\text{n}x}_{m}=1-\frac{{w}_{1}(1+\text{l}\text{n}{x}_{1})}{{w}_{1}+{w}_{2}{\lambda }_{12}}-\frac{{w}_{2}(1+\text{l}\text{n}{x}_{2})}{{w}_{1}{\lambda }_{21}+{w}_{2}}$$

${\lambda }_{12}$ and ${\lambda }_{21}$are the equation parameters.

### Model accuracy

The experimental solubility data were fitted using the previously mentioned equations, and mean relative deviation (MRD%) was employed as a measure of the model’s accuracy following the Eq. 

6 $$ \%MRD=\frac{100}{N}\sum (\frac{\left|Calculated\,value-Observed\,value\right|}{Observed\,value})$$

where *N* is the number of data points.

### Calculation of apparent thermodynamic parameters

A solute’s thermodynamic properties during the dissolution process can provide useful information regarding the solute’s behavior as it moves through a solvent mixture. The van’t Hoff analysis can be used to determine the apparent standard dissolution enthalpy for the dissolution of SA in mixtures of 2- propanol + EG and 2-propanol + PG.

7 $$ \frac{\partial \left(\text{l}\text{n} x\right)}{{\partial (\frac{1}{T}-\frac{1}{{T}_{hm}})}_{p}}=-\frac{{\Delta }H^\circ }{R}$$

The expression “*R*” denotes the universal gas constant with a precise numerical value of 8.314 JK^− 1^mol^− 1^. " *T*_hm_ " represents the mean harmonic temperature, which is obtained through Eq. (8).

8 $${T_{hm}}=\frac{N}{{\sum\limits_{{i=1}}^{N} {\frac{1}{{{T_i}}}} }}$$

The calculation of the standard Gibbs free energy of dissolution (ΔG˚) and the values of enthalpy ΔH˚ for the saturated mixed solutions can be done by determining the intercept and slope of the plot of ln *x*_1,T_ against $$ 1/T-1/{T}_{hm}$$. The Gibbs equation serves as a useful tool for computing the standard entropy of dissolution values (ΔS˚) [18]. Given that both entropy and enthalpy play a role in the process of dissolution, it is possible to represent their contributions using appropriate Eq. 

9 $$ {\varsigma }_{H}=\left(\frac{\left|{{\Delta }H}^{^\circ }\right|}{\left|{{\Delta }H}^{^\circ }\right|+\left|{T{\Delta }S}^{^\circ }\right|}\right)$$

10 $$ {\varsigma }_{TS}=\frac{\left|T{{\Delta }S}^{^\circ }\right|}{\left|{\Delta }{H}^{^\circ }\right|+\left|T\varDelta {S}^{^\circ }\right|}$$

## Results and discussion

### Reliability proof of the experimental method

**Fig. 2:**
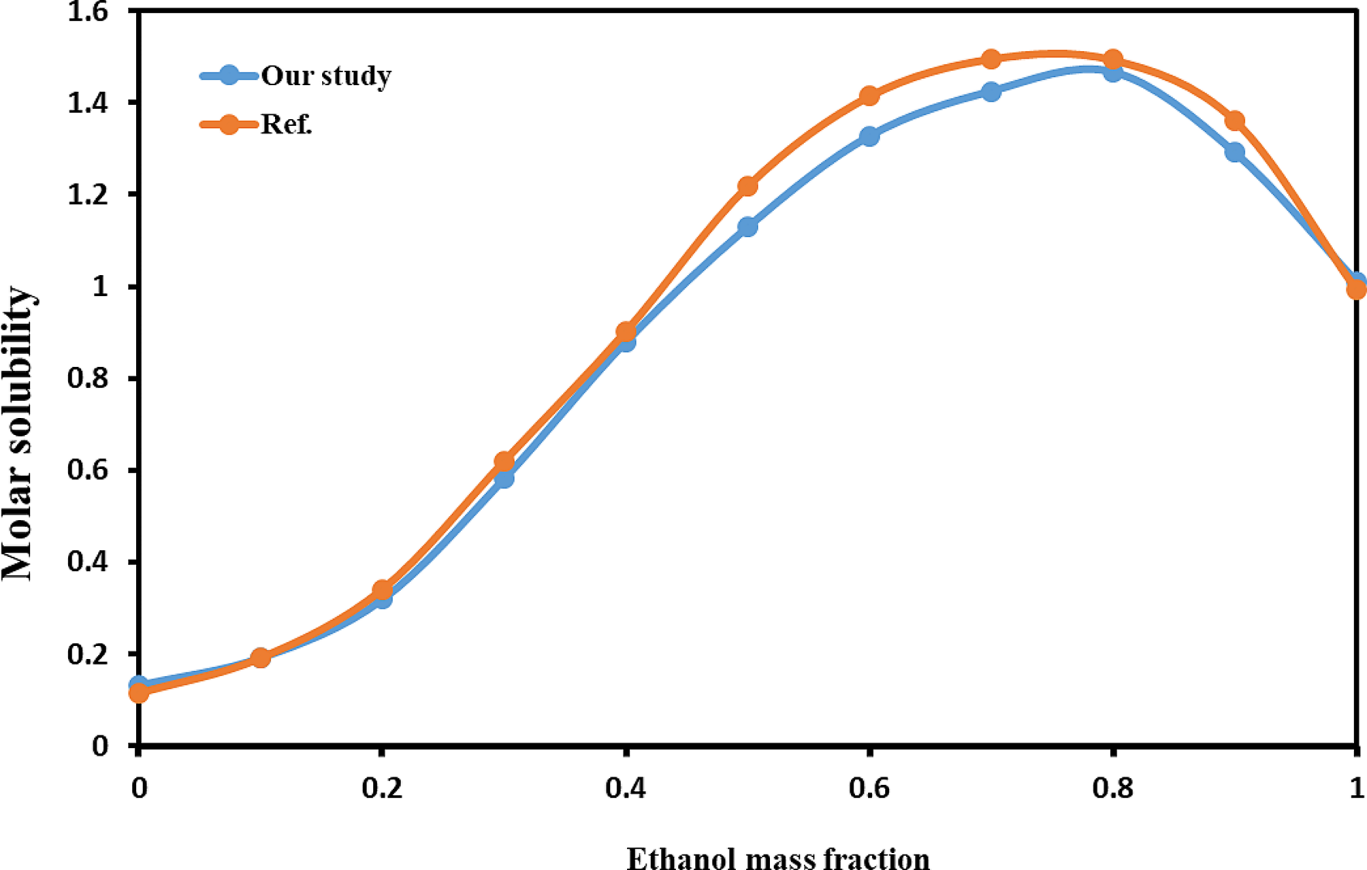
Molar solubility data of acetaminophen in ethanol + water mixture at 303.2 K for comparison with literature data

By quantifying the solubility of acetaminophen in an ethanol-water mixture through the available experimental setup and contrasting the findings with the referenced data [19], the method’s dependability and the apparatus’ precision were validated. As demonstrated in Fig. 2, the disparity in solubility between the literature value and the current study’s measurements was less than 10%, confirming the method’s and apparatus’ reliability.

### XRD analysis

**Fig. 3:**
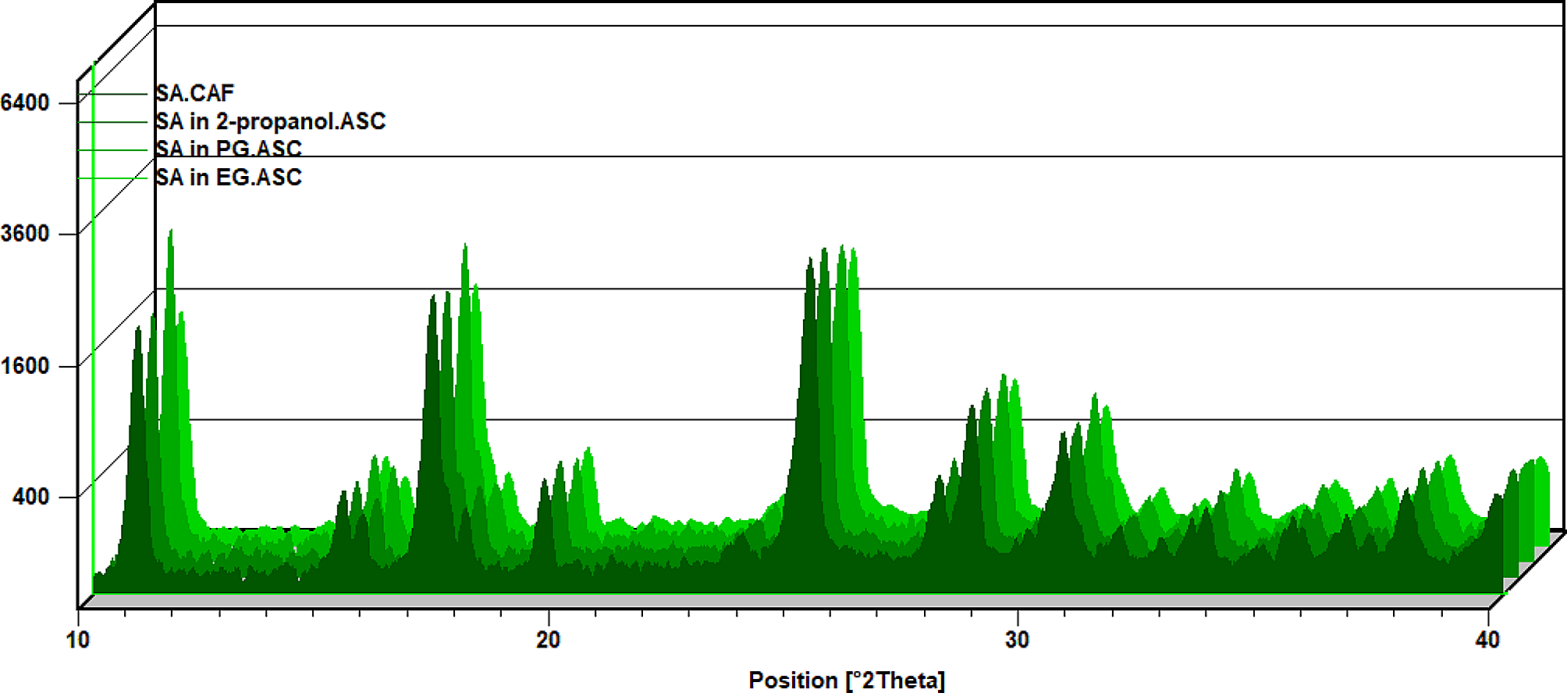
XRD pattern of raw SA and equilibrated SA in EG, PG and 2-propanol

Through XRD analysis at ambient temperature and pressure, the XRD data of SA residuals in individual solvents were acquired and depicted in Fig. 3. This examination determined whether solid SA in saturated solutions formed solvated compounds or polymorphs. Notably, no new characteristic peaks emerged, indicating that SA’s crystallinity remained consistent and did not undergo polymorphic transformation during the dissolution process.

### Solubility of SA in the binary mixtures

The experimental solubility data for SA in mixtures of (2-propanol + PG) and (2-propanol + EG) at five different temperatures were presented in Table 1. The reported values were the mean results obtained from three experiments, with the standard deviation (SD) indicated in parentheses. In both solvent systems, it can be observed that as the temperature increases and the concentration of 2-propanol increases, the solubility of SA also increases. This trend is evident from the higher solubility values observed at higher temperatures and 2-propanol mass fraction of 0.7 for (2-propanol + PG) binary mixtures and 0.4 for (2-propanol + EG) binary mixture. In mixtures containing both systems, the lowest solubility of SA occurred at the lowest concentration of the 2-propanol (w_1_ = 0.0, or neat PG or EG). Specifically, in the (2-propanol + PG) system at 298.2 K, the mole fraction solubility of SA was 9.36 × 10^− 2^ at neat PG. As the concentration of 2-propanol increased, the solubility of SA also increased steadily, reaching a maximum value of 1.68 × 10^− 1^ at w_1_ = 0.7. This maximum solubility represents the highest amount of SA that can be dissolved in this specified mixture under the given experimental conditions. A similar trend was observed in the (2-propanol + EG) system, with the lowest solubility of SA occurring at the lowest concentration of 2-propanol (neat EG). As the concentration of 2-propanol increased, the solubility of SA also increased gradually, reaching a maximum value (for example 2.39 × 10^− 1^ at 298.2 K) at *w*_1_ = 0.4 before decreasing with subsequent increases in 2-propanol concentration.

Considering that SA with log *P* = 2.26 [20], and dipole moment: 2.65 D [21], EG with log *P* = -1.69, dipole moment: 2.27 D, and dielectric constant of 41.2 [22], PG with log *P* = -0.92, and dipole moment: 2.27 D and dielectric constant of 32 [23], and 2-propanol with log *P* = -0.19, dipole moment: 1.66 D, and dielectric constant of 19.92 [24], it was expected that the solubility of SA would rise with the incorporation of 2-propanol, which is less polar than EG and PG. The possible reason can be explained by considering the overall polarity and solubility characteristics of the solvents involved. While EG and PG have a higher dielectric constant compared to 2-propanol, which suggests a greater ability to dissolve polar solutes, such as SA, other factors need to be taken into account. SA has a log *P* value of 2.26, indicating a moderate level of lipophilicity and a preference for non-polar environments. 2-Propanol is less polar overall when considering factors such as dipole moment and log *P*. This lower polarity makes 2-propanol a more suitable solvent for a moderately lipophilic compound like SA. This prediction was consistent with the findings observed in the experiments.

**Fig. 4:**
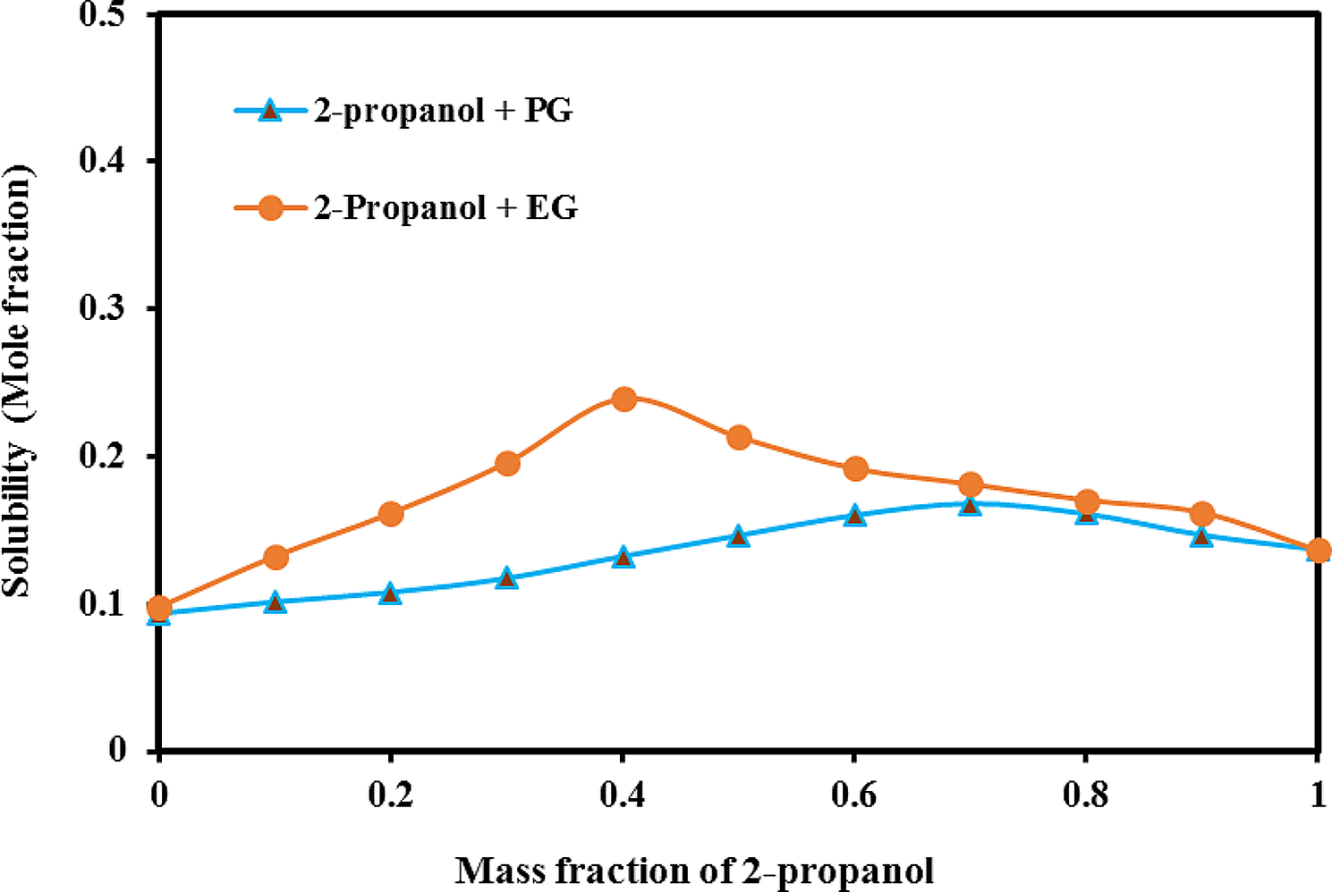
Mole fraction solubility of SA in both cosolvent mixtures at 298.2 K

Furthermore, Table 1; Fig. 4 showed the solubility of SA was relatively greater in (2-propanol + EG) than in (2-propanol + PG) under nearly all examined conditions demonstrating mixtures of 2-propanol + EG were more compatible solvents than mixtures of 2-propanol and PG for SA dissolution.

**Table 1:** Experimental mole fraction solubility (*x*_m, T_) as the average of three measured for SA in the binary (2-propanol + PG) and (2-propanol + EG) at *T* = 293.15 to 313.15 K and atmospheric pressure (≈ 85 kPa)

w_1_^a^	293.2 K	298.2 K	303.2 K	308.2 K	313.2 K
2-Propanol + EG					
0.00	8.71 (± 0.41) × 10^− 2^	9.80 (± 0.35) × 10^− 2^	1.10 (± 0.05) × 10^− 1^	1.18 (± 0.02) × 10^− 1^	1.33 (± 0.02) × 10^− 1^
0.10	1.11 (± 0.06) × 10^− 1^	1.32(± 0.06) ×10^− 1^	1.46 (± 0.00) × 10^− 1^	1.59 (± 0.05) × 10^− 1^	1.75 (± 0.07) × 10^− 1^
0.20	1.46 (± 0.05) × 10^− 1^	1.61 (± 0.04) × 10^− 1^	1.83 (± 0.01) × 10^− 1^	2.06 (± 0.10) × 10^− 1^	2.21 (± 0.04) × 10^− 1^
0.30	1.76 (± 0.01) × 10^− 1^	1.95 (± 0.14) × 10^− 1^	2.26 (± 0.14) × 10^− 1^	2.54 (± 0.12) × 10^− 1^	2.78 (± 0.14) × 10^− 1^
0.40	2.08 (± 0.07) × 10^− 1^	2.39 (± 0.15) × 10^− 1^	2.67 (± 0.17) × 10^− 1^	2.99 (± 0.00) × 10^− 1^	3.21 (± 0.08) × 10^− 1^
0.50	1.92 (± 0.06) × 10^− 1^	2.13 (± 0.06) × 10^− 1^	2.44 (± 0.09) × 10^− 1^	2.76 (± 0.03) × 10^− 1^	3.01 (± 0.19) × 10^− 1^
0.60	1.71 (± 0.08) × 10^− 1^	1.91 (± 0.06) × 10^− 1^	2.22 (± 0.09) × 10^− 1^	2.47 (± 0.14) × 10^− 1^	2.73 (± 0.17) × 10^− 1^
0.70	1.60 (± 0.02) × 10^− 1^	1.81 (± 0.10) × 10^− 1^	2.08 (± 0.07) × 10^− 1^	2.26 (± 0.10) × 10^− 1^	2.51 (± 0.19) × 10^− 1^
0.80	1.55 (± 0.02) × 10^− 1^	1.70 (± 0.06) × 10^− 1^	1.90 (± 0.08) × 10^− 1^	2.05 (± 0.07) × 10^− 1^	2.21 (± 0.19) × 10^− 1^
0.90	1.45 (± 0.00) × 10^− 1^	1.62 (± 0.10) × 10^− 1^	1.78 (± 0.13) × 10^− 1^	1.91 (± 0.08) × 10^− 1^	2.06 (± 0.16) × 10^− 1^
1.00	1.23 (± 0.05) × 10^− 1^	1.36 (± 0.07) × 10^− 1^	1.54 (± 0.12) × 10^− 1^	1.64 (± 0.11) × 10^− 1^	1.78 (± 0.01) × 10^− 1^
2-Propanol + PG					
0.00	8.98 (± 0.28) × 10^− 2^	9.36 (± 0.34) × 10^− 2^	9.73 (± 0.60) × 10^− 2^	9.85 (± 0.16) × 10^− 2^	1.02 (± 0.03) × 10^− 1^
0.10	9.66 (± 0.46) × 10^− 2^	1.01(± 0.02) × 10^− 1^	1.05 (± 0.01) × 10^− 1^	1.06 (± 0.02) × 10^− 1^	1.09 (± 0.05) × 10^− 1^
0.20	1.03 (± 0.04) × 10^− 1^	1.08 (± 0.02) × 10^− 1^	1.14 (± 0.02) × 10^− 1^	1.15 (± 0.05) × 10^− 1^	1.17 (± 0.04) × 10^− 1^
0.30	1.12 (± 0.04) × 10^− 1^	1.17 (± 0.03) × 10^− 1^	1.23 (± 0.04) × 10^− 1^	1.23 (± 0.01) × 10^− 1^	1.26 (± 0.00) × 10^− 1^
0.40	1.27 (± 0.04) × 10^− 1^	1.32 (± 0.06) × 10^− 1^	1.40 (± 0.04) × 10^− 1^	1.39 (± 0.04) × 10^− 1^	1.45 (± 0.04) × 10^− 1^
0.50	1.41 (± 0.02) × 10^− 1^	1.46 (± 0.02) × 10^− 1^	1.56 (± 0.03) × 10^− 1^	1.55 (± 0.02) × 10^− 1^	1.60 (± 0.05) × 10^− 1^
0.60	1.54 (± 0.09) × 10^− 1^	1.60 (± 0.05) × 10^− 1^	1.69 (± 0.07) × 10^− 1^	1.68 (± 0.04) × 10^− 1^	1.73 (± 0.04) × 10^− 1^
0.70	1.60 (± 0.05) × 10^− 1^	1.68 (± 0.10) × 10^− 1^	1.77 (± 0.02) × 10^− 1^	1.76 (± 0.02) × 10^− 1^	1.81 (± 0.03) × 10^− 1^
0.80	1.54 (± 0.10) × 10^− 1^	1.61 (± 0.02) × 10^− 1^	1.69 (± 0.05) × 10^− 1^	1.69 (± 0.03) × 10^− 1^	1.75 (± 0.08) × 10^− 1^
0.90	1.42 (± 0.03) × 10^− 1^	1.46 (± 0.08) × 10^− 1^	1.58 (± 0.05) × 10^− 1^	1.58 (± 0.10) × 10^− 1^	1.63 (± 0.03) × 10^− 1^
1.00	1.29 (± 0.04) × 10^− 1^	1.37 (± 0.01) × 10^− 1^	1.47 (± 0.04) × 10^− 1^	1.46 (± 0.07) × 10^− 1^	1.53 (± 0.05) × 10^− 1^

^a^*w*_1_ is the mass fraction of 2-propanol in the investigated mixtures in the absence of SA

### Solubility data modeling

The five correlative models including the van’t Hoff, Jouyban-Acree, Jouyban-Acree-van’t Hoff, MRS and the modified Wilson were used to represent the SA solubility data in (2-propanol + PG) and (2-propanol + EG) as mathematical models. Parameter of each model along with the *MRDs*% for back-calculated data was summarized in Tables 2, 3, 4 and 5.

**Table 2:** The van’t Hoff model parameters and the corresponding *MRD*% for SA in two investigated binary mixtures

w_1_		2-Propanol + EG			2-Propanol + PG			
	A	B	MRD%		A	B	MRD%	
0.00	4.096	-1914.749	1.0		-0.511	-554.973	0.6	
0.10	4.735	-2024.124	1.8		-0.472	-543.734	0.9	
0.20	4.765	-1960.746	1.0		-0.268	-583.904	0.9	
0.30	5.574	-2143.669	1.0		-0.360	-532.810	1.2	
0.40	5.283	-2004.347	1.3		-0.49	-588.414	1.0	
0.50	5.566	-2116.554	0.9		0.044	-584.849	1.1	
0.60	5.704	-2189.974	0.9		-0.115	-511.181	1.0	
0.70	5.239	-2070.577	0.8		-0.014	-528.003	1.2	
0.80	3.730	-1638.486	0.6		0.011	-547.738	0.9	
0.90	3.497	-1587.352	0.8		0.303	-659.591	1.1	
1.00	3.695	-1694.555	1.0		0.522	-748.821	1.2	
Overall MRD%			1.0				1.0	

**Table 3:** The parameters of the Jouyban-Acree, Jouyban-Acree-van’t Hoff and the corresponding *MRDs*% for SA in two binary investigated mixtures

	Jouyban-Acree		Jouyban-Acree-van’t Hoff	
2-Propanol + EG	*J*_0_	784.579	*A*_1_	3.695
	*J*_1_	-326.921	*B*_1_	-1694.555
	*J*_2_	-172.964	*A*_2_	4.096
			*B*_2_	-1914.749
			*J*_0_	784.344
			*J*_1_	-326.805
			*J*_2_	-173.553
***MRD*** **%**	3.7		3.8	
	Jouyban-Acree		Jouyban-Acree-van’t Hoff	
2-Propanol + PG	*J*_0_	316.757	*A*_1_	0.522
	*J*_1_	278.020	*B*_1_	-748.821
	*J*_2_	-94.941	*A*_2_	-0.511
			*B*_2_	-554.973
			*J*_0_	316.985
			*J*_1_	277.961
			*J*_2_	-94.371
***MRD*** **%**	1.3		1.7	

**Table 4:** The MRS model constants at the investigated temperatures and the corresponding *MRDs*% for SA in two binary investigated mixtures

Binary system	T (K)	β_1_	β_2_	β_3_		β_4_		β_5_		MRD%
2-Propanol + EG	293.2	-2.206	-2.452	0^a^		0^a^		2.666		6.6
	298.2	-2.104	-2.317	0^a^		0^a^		2.654		5.9
	303.2	-1.999	-2.209	0^a^		0^a^		2.757		5.9
	3.8.2	-2.164	-2.184	0^a^		0.006		3.524		4.8
	313.2	-2.067	-2.076	0^a^		0.006		3.443		4.2
	**Overall** ***MRD*** **%**									**5.5**
2-Propanol + PG	293.2	-2.034	-2.686	0^a^		0.005		1.613		3.4
	298.2	-1.985	-2.625	0^a^		0.004		1.558		3.4
	303.2	-1.916	-2.581	0^a^		0.004		1.554		2.9
	3.8.2	-1.915	-2.568	0^a^		0.004		1.505		2.9
	313.2	-1.872	-2.533	0^a^		0.004		1.466		2.8
	**Overall** ***MRD*** **%**									**3.1**

**Table 5:** The modified Wilson Model parameters at the investigated temperatures and the corresponding *MRDs*% for SA in two binary investigated mixtures

T (K)	2-Propanol + EG				2-Propanol + PG		
	λ_12_	λ_21_	MRD%		λ_12_	λ_21_	MRD%
293.2	1.868	3.607	4.9		3.858	0.840	2.7
298.2	2.069	4.120	4.6		3.687	0.882	2.6
303.2	2.467	4.692	5.0		3.746	0.929	2.6
308.2	3.002	5.858	5.8		3.774	0.895	2.7
313.2	3.620	6.293	6.4		3.789	0.902	2.6
Overall *MRD*%			5.3				2.6

Table 2 displays the parameters acquired from the van’t Hoff model as well as their corresponding MRDs% for SA in the investigated mixtures of (2-propanol + EG) and (2-propanol + PG). The parameters *A* and *B* represent the intercept and slope of the van’t Hoff equation, respectively. The *MRD*% values indicate the accuracy of the model predictions compared to the experimental data. In both mixtures, the *MRD*% values range from 0.6 to 1.8%, suggesting a good agreement between the van’t Hoff model and the experimental solubility data. Table 3 presents the calculated parameters for the Jouyban-Acree and Jouyban-Acree-van’t Hoff models, illustrating SA solubility in the (2-propanol + EG) and (2-propanol + PG) mixtures. The parameters *J*_*0*_, *J*_*1*_, and *J*_*2*_ are specific to the Jouyban-Acree model, while *A*_*1*_, *B*_*1*_, *A*_*2*_, and *B*_*2*_ correspond to the Jouyban-Acree-van’t Hoff model. The *MRD*% values for both models in both mixtures range from 1.3 to 3.8%, indicating a reasonably good fit between the models and the experimental data. Table 4 presents the constants (*β*_*1*_-*β*_*5*_) obtained from the MRS model for SA solubility. The *MRD*% values range from 2.8 to 6.6%, suggesting a satisfactory agreement between the MRS model predictions and the experimental solubility data. Table 5 provides the modified Wilson model parameters (*λ*_*12*_ and *λ*_*21*_) for SA solubility at various temperatures. The MRD% values range from 2.6 to 6.4%, indicating a good fit between the modified Wilson model and the experimental solubility data. In general, the application of mathematical models shows promising results in predicting and describing the solubility of SA in the investigated mixtures. These models provide valuable insights into the dissolution process and offer reasonable accuracy in representing the experimental solubility data.

Apart from correlation analysis and back-calculation computations, the predictive ability of the Jouyban-Acree-van’t Hoff model as a semi-predictive model for solubility data was also evaluated. The model was trained using a limited number of data points, specifically the solubility data for mono-solvents at low and high temperatures, as well as solvent mixtures with mass fractions of 0.3, 0.5, and 0.7 at 298.2 K. The model was then employed to predict the remaining data for other mass fractions and temperatures. The prediction *MRDs*% for various temperatures of the (2-propanol + EG) system were 4.5%, 4.1%, 3.4%, 3.5%, and 3.6% at 293.2 K, 298.2 K, 303.2 K, 308.2 K, and 313.2 K, respectively. For the (2-propanol + PG) system, the prediction *MRDs*% at 293.2 K, 298.2 K, 303.2 K, 308.2 K, and 313.2 K were 1.6%, 1.5%, 2.4%, 1.5%, and 1.4%, respectively.

Table 6 displays the measured densities of SA-saturated solutions in the two investigated cosolvent mixtures at different temperatures, along with their corresponding SD. These density values offer crucial insights into the physical properties of SA solutions in binary mixtures, as they were obtained through direct measurements.

In addition to examining solubility data, adapted version of the Jouyban-Acree model was also employed to establish correlations with density values, leading to the development of the following trained equations for the models:

11 $$ \text{l}\text{n}{\rho }_{m,T}={w}_{1}\text{l}\text{n}{\rho }_{1,T}+{w}_{2}\text{l}\text{n}{\rho }_{2,T}-1.267\frac{{w}_{1}.{w}_{2}}{T}$$

12 $$\begin{array}{l}\text{l}\text{n}{\rho }_{m,T}\\={w}_{1}\text{l}\text{n}{\rho }_{1,T}+{w}_{2}\text{l}\text{n}{\rho }_{2,T}-5.851\frac{{w}_{1}.{w}_{2}}{T}\\-3.554\frac{{w}_{1}.{w}_{2}{ (w}_{1}-{w}_{2})}{T}\end{array}$$

where $\rho$_1,*T*_ and $\rho$_2,*T*_ are densities of the saturated mono-solvents 1 and 2, $\rho$_*m, T*_ is the drug density of the saturated -solvent mixture at temperature *T*. Equations (11) and (12) represent the trained models for density data of SA-saturated solutions in mixtures consisting of (2-propanol + EG) and (2-propanol + PG), respectively. The *MRD*% for the back-calculated data was determined to be 0.2% and 0.4% for Eqs. (11) and (12), respectively. These low *MRD*% values suggest that the Jouyban-Acree model was highly reliable for predicting density values in these binary mixtures.

**Table 6:** Measured density (g/cm^3^) of SA-saturated solutions in the investigated binary mixtures at different temperatures

w_1_	293.2 K	298.2 K	303.2 K	308.2 K	313.2 K
2-Propanol + EG					
0.00	1.132 ± 0.003	1.130 ± 0.003	1.127 ± 0.003	1.124 ± 0.003	1.112 ± 0.006
0.10	1.098 ± 0.001	1.095 ± 0.003	1.095 ± 0.003	1.095 ± 0.003	1.095 ± 0.008
0.20	1.076 ± 0.001	1.072 ± 0.001	1.071 ± 0.001	1.066 ± 0.001	1.066 ± 0.005
0.30	1.049 ± 0.001	1.047 ± 0.001	1.047 ± 0.005	1.041 ± 0.003	1.042 ± 0.005
0.40	1.029 ± 0.001	1.028 ± 0.001	1.021 ± 0.001	1.016 ± 0.001	1.016 ± 0.005
0.50	1.000 ± 0.003	1.000 ± 0.003	1.000 ± 0.003	0.995 ± 0.003	0.995 ± 0.005
0.60	0.980 ± 0.003	0.980 ± 0.003	0.974 ± 0.003	0.968 ± 0.003	0.968 ± 0.005
0.70	0.954 ± 0.001	0.953 ± 0.003	0.952 ± 0.003	0.951 ± 0.003	0.945 ± 0.005
0.80	0.932 ± 0.003	0.932 ± 0.001	0.930 ± 0.005	0.926 ± 0.005	0.926 ± 0.005
0.90	0.908 ± 0.003	0.908 ± 0.001	0.908 ± 0.005	0.908 ± 0.005	0.906 ± 0.005
1.00	0.887 ± 0.003	0.887 ± 0.001	0.884 ± 0.003	0.886 ± 0.003	0.885 ± 0.005
2-Propanol + PG					
0.00	1.071 ± 0.001	1.068 ± 0.003	1.058 ± 0.005	1.058 ± 0.001	1.058 ± 0.003
0.10	1.045 ± 0.003	1.037 ± 0.001	1.032 ± 0.003	1.032 ± 0.003	1.028 ± 0.001
0.20	1.027 ± 0.001	1.025 ± 0.003	1.025 ± 0.003	1.023 ± 0.003	1.023 ± 0.003
0.30	1.009 ± 0.003	0.997 ± 0.003	0987 ± 0.001	0.987 ± 0.005	0.987 ± 0.003
0.40	0.989 ± 0.001	0.986 ± 0.003	0.970 ± 0.005	0.970 ± 0.003	0.969 ± 0.001
0.50	0.976 ± 0.003	0.970 ± 0.003	0.948 ± 0.001	0.948 ± 0.003	0.941 ± 0.001
0.60	0.954 ± 0.003	0.953 ± 0.003	0.934 ± 0.003	0.934 ± 0.003	0.913 ± 0.003
0.70	0.940 ± 0.001	0.933 ± 0.001	0.913 ± 0.003	0.913 ± 0.003	0.901 ± 0.001
0.80	0.916 ± 0.003	0.910 ± 0.003	0.895 ± 0.003	0.892 ± 0.003	0.892 ± 0.003
0.90	0.896 ± 0.001	0.901 ± 0.001	0.877 ± 0.005	0.877 ± 0.003	0.873 ± 0.003
1.00	0.878 ± 0.003	0.887 ± 0.003	0.860 ± 0.005	0.860 ± 0.003	0.859 ± 0.003

### Calculation of apparent thermodynamic parameters

Table 7 presents a summary of the apparent thermodynamic functions that represent the dissolution of SA in mixtures consisting of (2-propanol + EG) and (2-propanol + PG) at a temperature of *T*_*hm*_ = 303.0 K. These functions were *ΔG*°, *ΔH*°, *ΔS*°, *TΔS*°, *ζ*_*H*_, and *ζ*_*TS*_. Positive values of *ΔG*° indicate that dissolution was not spontaneous, while positive values of *ΔH*° suggest that heat was absorbed during dissolution in an endothermic process. The values of *ΔS*° that were positive signify the advantageous contribution of entropy to the process of dissolution. Based on these thermodynamic parameters, it can be concluded that SA dissolves readily in mixtures of (2-propanol + EG) and (2-propanol + PG) by decreasing ΔG° with minimum value in the mixture which SA has high solubility, increasing entropy, and absorbing heat.

**Table 7:** Apparent thermodynamic parameters for SA dissolution behavior in the investigated binary mixtures at *T*_hm_ = 303.0 K

w_1_	ΔG° (kJ mol^− 1^)	ΔH° (kJ mol^− 1^)	ΔS° (J mol^− 1^ K^− 1^)	TΔS° (kJ mol^− 1^)	ζH	ζTS
EG + 2-Propanol						
0.00	5.60	15.92	34.05	10.32	0.607	0.393
0.10	4.90	16.83	39.37	11.93	0.585	0.415
0.20	4.30	16.30	39.62	12.00	0.576	0.424
0.30	3.78	17.82	46.34	14.04	0.559	0.441
0.40	3.36	16.66	43.92	13.31	0.556	0.444
0.50	3.58	17.60	46.28	14.02	0.557	0.443
0.60	3.84	18.21	47.42	14.37	0.559	0.441
0.70	4.02	17.21	43.56	13.20	0.566	0.434
0.80	4.23	13.62	31.01	9.40	0.592	0.408
0.90	4.39	13.20	29.08	8.81	0.600	0.400
1.00	4.78	14.09	30.72	9.31	0.602	0.398
PG + 2-Propanol						
0.00	5.90	4.61	-4.24	-1.29	0.782	0.218
0.10	5.71	4.52	-3.92	-1.19	0.792	0.208
0.20	5.53	4.85	-2.23	-0.68	0.878	0.122
0.30	5.34	4.43	-2.99	-0.91	0.830	0.170
0.40	5.02	4.89	-0.40	-0.12	0.975	0.025
0.50	4.75	4.86	0.36	0.11	0.978	0.022
0.60	4.54	4.25	-0.96	-0.29	0.936	0.064
0.70	4.43	4.39	-0.12	-0.04	0.992	0.008
0.80	4.53	4.55	0.09	0.03	0.994	0.006
0.90	4.72	5.48	2.52	0.76	0.878	0.122
1.00	4.91	6.23	4.34	1.32	0.826	0.174

**Fig. 5:**
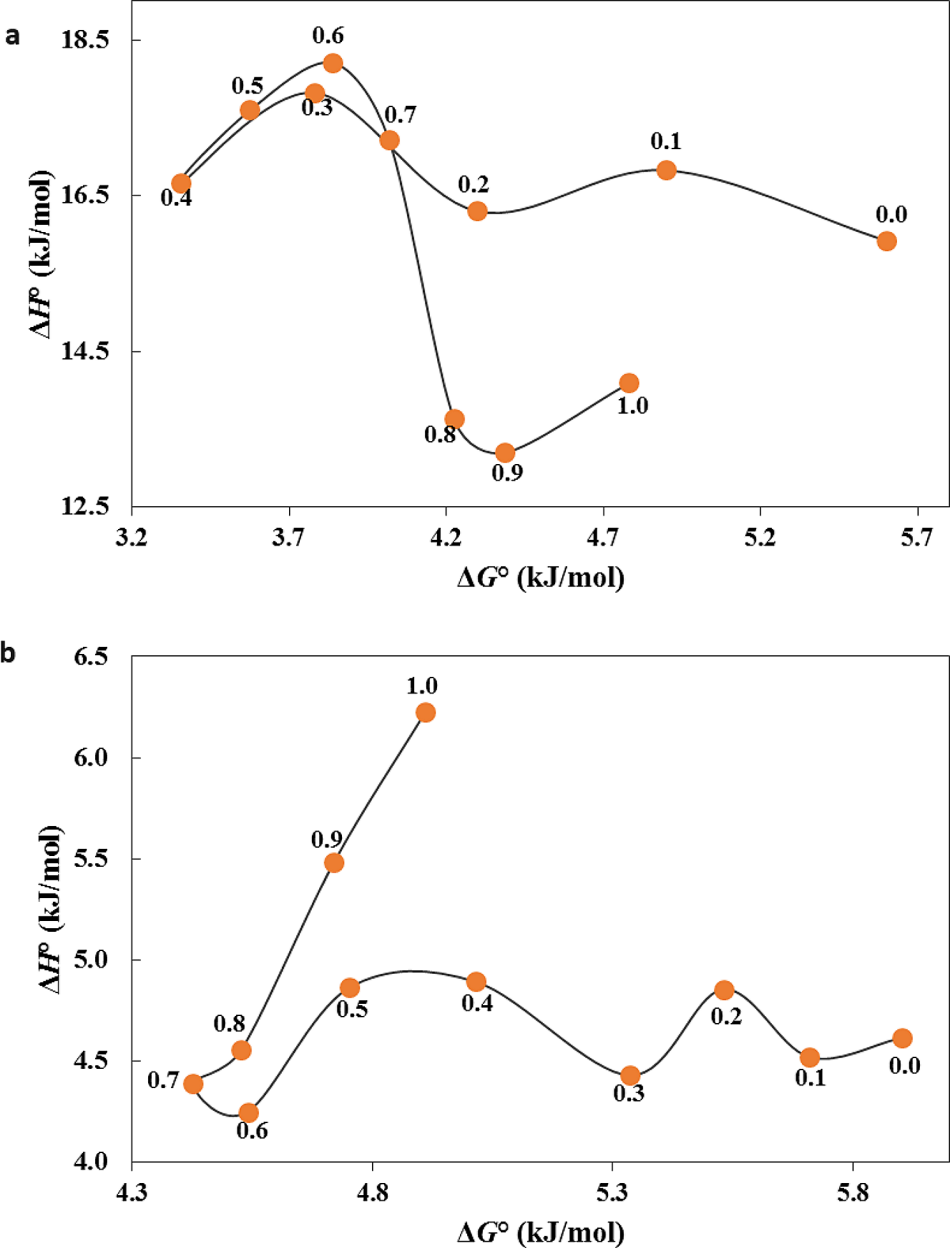
Enthalpy-entropy compensation plot for SA in **a**: (2-propanol + EG) and **b**: (2-propanol + PG) mixtures at *T*_hm_ = 303.0 K. The points present the mass fractions of 2-propanol in the investigated mixtures in the absence of SA

In the case of SA dissolution, the enthalpy-entropy compensation analysis can help determine whether changes in enthalpy and entropy were correlated. By plotting the values of *ΔH°* and *ΔG°* for different compositions of binary mixtures, it is possible to analyze the relationship between enthalpy and entropy changes. The enthalpy-entropy compensation plot for (2-propanol + EG) shown in Fig. 5a reveals that some linear correlation lines with different slopes fit the data. This suggests that both enthalpy-driven and entropy-driven processes contribute to SA solubility. In mixtures with 0.1 ≤ *w*_1_ ≤ 0.2, 0.3 ≤ *w*_1_ ≤ 0.6, and 0.9 ≤ *w*_1_ ≤ 1.0 the plots showed a positive slope, indicating that the transfer of SA in these mixtures was primarily driven by enthalpy effects. The decrease in enthalpy was accompanied by a corresponding decrease in free energy, suggesting that the transfer process in these mixtures was mainly influenced by enthalpic interactions between SA and the solvent components. For mixtures with 0.0 ≤ *w*_1_ ≤ 0.1, 0.2 ≤ *w*_1_ ≤ 0.3 and 0.6 ≤ *w*_1_ ≤ 0.9 the plots showed a negative slope, suggesting that the solubility of SA in these mixtures was predominantly influenced by entropy effects. The decrease in *ΔG°* was accompanied by an increase in *ΔH°*, implying that in these mixtures, the dissolution process was forced by entropic factors such as increased disorder or solvation effects. For the (2-propanol + PG) mixture, enthalpy-entropy compensation plot was illustrated in Fig. 5b. The curve exhibits a negative slope for mixtures 0.1 ≤ *w*_1_ ≤ 0.2, 0.3 ≤ *w*_1_ ≤ 0.5 and 0.6 ≤ *w*_1_ ≤ 0.7 indicating that entropy effects were the main factor affecting the solubility of SA in these mixtures. A positive slope for mixtures 0.0 ≤ *w*_1_ ≤ 0.1, 0.2 ≤ *w*_1_ ≤ 0.3, 0.5 ≤ *w*_1_ ≤ 0.6 and 0.8 ≤ *w*_1_ ≤ 1.0 was observed, indicating that enthalpy effects were the main factor affecting the solubility of SA in these mixtures. Overall, the enthalpy-entropy compensation plot demonstrates that the solubility of SA was influenced by both enthalpic and entropic contributions, with different driving forces depending on the composition of the solvent mixtures.

## Conclusion

In this study, the solubility and thermodynamics of SA in binary solvent mixtures of (2-propanol + EG) and (2-propanol + PG) at different temperatures in the range of 293.2–313.2 K were measured using a shake-flask method followed by spectrophotometery measurment. It was found that SA became more soluble with increasing alcohol concentration and temperature in both solvent systems. In addition, it was observed that the solubility of SA was higher in the combination of 2-propanol and EG compared to the mixture of 2-propanol and PG in most tested situations. This suggests that 2-propanol and EG mixtures were more suitable solvents for dissolving SA than those consisting of 2-propanol and PG. Soem mathematical models were used in this study to correlate the solubility data and obtained the *MRDs*% of 0.6–1.8% for van’t Hoff model, 1.3–3.8% for Jouyban-Acree, Jouyban-Acree-van’t Hoff models, 2.8–6.6% for MRS mode, and 2.6–6.4% for modified Wilson model for back-calculated data showed SA solubility was accurately predicted using these models with *MRDs*% less than 7.0%. Furthermore, various thermodynamic properties such as *ΔG°*, *ΔH°*, and *ΔS°* were derived from the experimental data using the van’t Hoff equation. This analysis indicated that SA dissolution in the studied solvent mixtures was a non-spenteneos, endothermic and entropy favor process.

## Data Availability

The datasets used and/or analysed during the current study are available from the corresponding author on reasonable request.
